# Ninth International Medical Congress, Washington, D. C.

**Published:** 1887-10

**Authors:** 


(TO BE CONTINUED.)

---

### NINTH INTERNATIONAL MEDICAL CONGRESS, WASHINGTON, D. C., SEPTEMBER, 1887.

### SECTION XVIII, DENTAL AND ORAL SURGERY.

REPORTED FOR THE INDEPENDENT PRACTITIONER BY "MRS. M. W. J."

The sessions of Section XVIII were held in the Universalist Church, corner 13th and L Streets.

The Section was called to order in first working session at 3.30 P. M., Monday, September 5th, by Prof. J. Taft, of Cincinnati, President of the Section.

Short addresses of welcome were delivered by Drs. Thackston and W. H. Morgan, and responses upon the part of the foreign visitors were made by Drs. McLeod, of Edinburgh; Metnitz, of Vienna; Sjolberg, of Stockholm, and Grevers, of Amsterdam.

The President, Dr. Taft, then delivered his opening address, taking for his topic

THE FACTORS AND FORCES IN THE DEVELOPMENT OF DENTISTRY.

He said that one hundred years ago the germ from which has grown our art and science had no special significance—apparently no soil in which to grow. It was attracting no attention from the public. Its literature was vague and desultory until the work of John Hunter, on The Natural History of the Human Teeth, in

1771, attracted general attention and met with full appreciation. Until within the last fifty years there were not more than from thirty to fifty volumes worthy of attention. In 1839 the work of S. Fitch, on Dental Surgery, appeared, and in less than ten years after more than twenty new books were published, some of which are still accepted as standard text-books. The appearance of the *American Journal of Dental Science,* in 1839, was the beginning of the journalistic literature of the world. Since then more than a hundred periodicals have been established. From that time dental journals have continued to increase their influence and power, and are more potent to-day than ever before. In 1840, the American Society of Dental Surgeons was organized, the pioneer in association work. This was disbanded after a prosperous career of sixteen years, but the Virginia State Society, founded in 1842, the Mississippi Valley Society, in 1844, the Pennsylvania Society of Dental Surgeons, in 1845, the New York State Society, in 1847, and many others founded at that early date, still survive in active usefulness. The American Dental Convention was organized in 1855, and the American Dental Association in 1859. There are now but few towns of a thousand inhabitants which have not their local societies. No man with any professional ambition can afford to stand aloof, and refuse to participate in this work. England, France and Germany have efficient societies, while the Odontological Society of Great Britain has not been excelled, if equaled. Its systematic and valuable work is shown in the twenty-five published volumes of transactions.

In 1840 the Baltimore College of Dental Surgery was founded, the first institution in the history of the world for special instruction in the science of dentistry. The proposition to establish a dental college was a bold one, but it was the only thing to be done, for medical colleges had been appealed to in vain. It required courage and ability beyond those of any ordinary man, for there were no antecedents. Arduous labor was inevitable, the prospects of emolument hopeless. It is very doubtful whether their most radiant visions ever reached the realization of to-day! In five years success was a demonstrated fact. There are now twenty-two recognized dental colleges. In Europe, the University of Leipsic was the first to establish a dental department. That of Berlin is more like our own university dental departments.

In the United States, thirty-six States and Territories now have laws regulating the practice of dentistry. The laws of Great Britain, Germany, France and other European countries, are also quite stringent on the subject.

These are some of the factors and forces that have been instrumental in developing dentistry. Are they exhausted ? Have they done all of which they are capable ? Have they reached their maximum ? We believe they are capable of doing far more. These forces are in better condition than ever before for effective labor. Never has literature made a better record, or been more appreciated or utilized. Association work has never been more highly appreciated. Our colleges have received fresh impetus on their onward and upward career. We are warranted in anticipating still higher results, for the outreach was never so active. In the dental profession there is unity ; there are no *isms*, no *pathies ;* we move forward in solid column, each individual an independent thinker, but all in common sympathy. With no factions or divisions on which to waste our strength, we press forward to the fulfillment of the highest interests before us. An expectation is abroad for the work to be done in this Section. Let us fill these expectations to the full.

At the conclusion of the President's address, the State Dental Society of Minnesota presented him with an emblematic gavel, in the shape of a mammoth molar, of white cement set in black rubber, with gold bands—the rubber emblematic of elasticity of thought, the gold of purity of motives, the cement of the ties that bind all members of the profession together—the whole representing something that is in everybody's mouth, and that it pains us to part with.

The President received the gavel with thanks, predicting that all would now go smoothly, and that if any one was out of order he would yield to the pronunciamento of such a gavel.

Dr. R. Finley Hunt wished to present to the Section his finished work in the shape of the arrangements made for their accommodation. Its success must be judged by themselves. He hoped a vote of thanks would be tendered the Commissioners of the District of Columbia, and the Board of School Managers, for the use of the Franklin school building for clinics and the exhibition of the dental manufactures. He did not include the church building, because that was paid for. He hoped that all the officers connected with the school buildings would be invited to attend the clinics and meetings. The vote was passed, and also a vote of thanks for the ample accommodations provided.

On motion of Dr. Marshall, of Chicago, a vote of thanks was tendered the President for his able and valuable address.

Dr. R. J. Porre, of Cincinnati, then read a paper entitled

"CHRONIC PYÆMIA OF DENTAL ORIGIN."

This was the history of a case of blood-poisoning of thirty years' standing, cured by the extraction of a suppurating impacted wisdom tooth. The patient had suffered in all the forms known to chronic pyæmia—eruptions with stubborn ulceration in hands and feet, frequent and painful inflammation of the throat, the bowels either constipated or distressingly loose, night sweats, rigors, pains in all parts of the body, darting and shifting from the maxilla to remote portions of the body. He had been treated for every local lesion, and for twenty years for syphilis. These are severe results to spring from one tooth, but they are morbid effects of animal poison in the system; of the distribution of morbific matter in the different tissues, heat, vertigo and rigors being modified by the supply of virus. Through changes induced by ulceration from inoculation, prostration and death may close the scene, or typhoid symptoms may intervene, or it may be either modified or aggravated by the intervention of other diseases, as scarlatina, diphtheria, etc. Nothing is more usual than discharges from the nose, ears, etc., or boils and abscesses in parts remote from the source of origin. All these pathological changes may follow the decomposition of animal tissue. The strength of the most powerful piece of machinery is only equal to that of its weakest part. A wandering corpuscle from supperating tissue, getting entangled in some debilitated part, begins its work of generation, and boils or carbuncles result. Pus itself is not the cause till it is demoralized by microbes, or ptomaines.

It is well understood that the gravity of the result depends on the vigor or vitality—the ability to cast out—and idiosyncrasies of the patient. Morbid secretions are capable of vitiating other territories. Scurvy is the cause of various lesions. Pyorrhœa alveolaris is another local lesion which is the source of the gravest results. Its etiology is not yet settled, whether local or constitutional, and it is very variable in its forms of development. As the results of diseases of the periosteum, vascular tissues and smaller nerves and vessels atrophy, and the larger ones are congested. With proliferation of germs the teeth loosen and suppuration begins in a little stream of pus, not sufficient to endanger life, but enough to cause a degree of suffering not commensurate with the apparent magnitude of the cause.

In the case of a patient of thirty years of age, with no symptoms of constitutional disease, there had been four repetitions of severe swelling and inflammation of the face and neck. Finding no local cause, it was believed to be malignant. When the cyst was opened a teaspoonful of yellow pus was discharged. The first and second bicuspids were missing, the three molars good, with no evidence of any remaining roots. More thorough, systematic search, however, revealed the roots of the first bicuspid. The stench was intolerable when they were removed, but the exudation was small and cheesy. It was treated with a mixture of carbolic acid and chloroform, each one drachm, and eucalyptus oil one scruple, and the patient sent to a physician to recuperate his general health, Here there had been the gravest misapprehension both in previous diagnosis and treatment.

In the next case, a young merchant of twenty-eight years of age was suffering severe pain in the right side of the face and eye. He was of good physical ancestry, and had had good health himself till the last eighteen months; he had no appetite, his nervous system was prostrated. Sea bathing, etc., had been tried in vain. The facial pains affecting the right eye, he had also been under the care of an oculist. There was a typhoid tone and impaired digestion. An alveolar abscess was found covering one-half the roof of the mouth. There was a leaking plug in the bicuspid, but the pulp was alive. Fluctuation in the abscess was plain, but the outlet was obscure. The gum was found slightly detached at the right superior bicuspid, and on pressure there was an exudation of sanious fluid. A free opening being made, there was an immense discharge of purulent matter, forced out by pressure on the sac. The cause was caries of the alveolus. It was successfully treated through the opening made, and did not return.

In the third case, there was traumatic lesion of the tongue, diagnosed as pyæmia. The patient was sixty-eight years of age, and of good physical history. There had been a gradual failure of health, of typhoid character, with cough and expectoration. Two teeth were suppurating, and the third lower molar cut into the tongue, distributing noxious matter by inoculation. Extraction afforded prompt cure.

The fourth case was also of good physical history, but had been suffering protean tortures for years, with every functional disorder. One peculiarity of pyæmia is its capricious tendencies and inexplicable ways. In this case there were furuncles of the scalp from cysts burrowing under the scalp. The extraction of ten bad teeth gave a complete cure.

In the fifth case, there was severe inflammation of the throat, which had been treated by a prominent physician for years as a disease of the throat, larynx and fauces. After the extraction of several molars the patient got well, the root of the third lower molar having been the special cause of the trouble.

The sixth case was facial paralysis. The pain was so severe that a naturally robust constitution gave way. The suffering was most intense in the left eye, which protruded frightfully. The paralysis began with a falling of the lips, which soon implicated the entire side of the face, paralysis being complete in four days. He was reduced in flesh to a mere shadow of himself, with various functional disturbances. There was a large filled cavity in the left lower second molar, the only tender tooth. The feeble condition of the patient forbade extraction, but the filling was removed, followed by an exudation of not uncertain odor, the result of chemical decomposition, putrefaction and absorption. Chloral, gum camphor, eucalyptus, alcohol and water were used in the treatment, and the improvement was phenomenal in the course of three or four days. The cure was radical, the patient's health being restored, the tooth saved and the paralysis cured.

Case seven was a representative case, the patient, sixty-four years of age. For four or five years he had been under medical treatment without avail, the cause not being found. Recognizing the symptoms of pyæmia, ulcerated teeth and gums were found, and seventeen teeth and roots were extracted. In ten days the patient had gained six pounds, and he progressed steadily to health.

In case eight, there was extensive caries and necrosis of the alveolus of the left maxilla, from the central incisor to the second bicuspid. There was an opening through the gums to the alveolus the size of a filbert. The abscess had spontaneously opened with a continuous sanious discharge, very offensive, but giving no pain. There had been no improvement for a year. Extracting three teeth, the necrosed bone and septum was cut away, and sulphuric acid injected into the sinus. In a week there was a favorable change, followed by restoration of bone in thirty days, and speedy restoration to health.

In case nine, there was serious general debility with uterine complications, the patient having been an invalid for years. Her station in life was such that she was obliged to give some attention to household duties, after which she would be obliged to seek her couch from extreme weakness. A painful condition of the teeth and mouth was found, necessitating the extraction of fifteen teeth and roots. The alveolus was also involved in caries and necrosis. The extraction was deferred for two or three months, lotions being used for the gums, when the operation was performed. The shock to the nervous system was much less than anticipated, and was followed by restored health. The uterus is the most sympathetic of all organs, becoming readily involved in ulceration and congestion. In uterine troubles, where no cause is apparent, the teeth should be looked to.

A word in behalf of the little ones. In the teeth we find the frequent cause of their diseases. In the majority of cases, from three to twelve years of age, the teeth are more or less carious. Pyæmia prevails with them as with adults, with less vital force to eliminate it, giving rise to most serious results. One collapsed abscess is sufficient to vitiate the tissues. Children are so susceptible to apparently trivial causes, that we find them panting their lives away, the cause unsuspected till the mouth is opened, and it is found in carious teeth and discharging abscesses.

Physicians are awakening to this fact, and are now sending ten cases to the dentist where they formerly sent one, and there are few cases which are more promptly relieved than chronic pyæmia of dental origin.

The discussion of Dr. Porre's paper was opened by Dr. J. Frank Lydston, of Chicago, and continued at the session of Tuesday, 11 A. M., by Drs. Geo. H. Chance, Portland, Oregon; W. C. Barrett, Buffalo, N. Y.; Mr. Walker, London, Eng.; A. O. Rawls, Lexington, Ky.; F. H. Rehwinkel, Chillicothe, Ohio; C. A. Brackett, Newport, R. I.; A. E. Baldwin, Chicago, Ill.; W. J. Younger, San Francisco, California; Genese, Baltimore; Whitefield, Evanston, Ill.; Story, Dallas, Texas; and others.

*Dr. Chance*—Exhibited a specimen of pyogenic membrane from a case in which the trouble arose from a left lateral incisor, with caries of the maxillary bone, almost reaching the palatal bone.

*Mr. Walker*—Expressed his surprise that Dr. Porre should so frequently use the forceps. According to his observations a great many cases were cured by treatment without extraction; in fact, it was the general rule in England.

*Dr. Rehwinkel*—Said that all were familiar with the pathology as described, but, as Prof. Dawson had once said to him, the idea of ascribing such serious results to a mere dental lesion was not long since an absurdity in the eyes of most physicians. The dentist who would have propounded such a thing to physicians five years ago, would have been called a fool for his pains. But now there are many physicians who send such cases to the dentist. The paper opens a large field for discussion, and in pyæmia, as in pyorrhœa, the dentist cannot confine himself to mere local treatment.

*Dr. Brackett*—Thought that more careful examination would reveal carious and necrosed bone far more frequently than was supposed.

*Dr. Younger*—Wished to suggest two remedies—nitric acid for the dissolution of necrosed bone, from its great affinity for lime-salts, and corrosive sublimate, one to four *per mille,* as a disinfectant.

*Dr. Genese*—Thought there was a possibility of harm from the caustic and escharotic action of the remedies proposed, and preferred dilute aromatic sulphuric acid as safer, defining the line of demarcation between living and dead bone without fear of bad effects. He said that a small pellet of the solid extract of white poppies applied to the affected part would afford relief. It could also be dissolved in water and applied externally as a wash.

*Dr. Whitefield*—Advised electrolytic action for the destruction of microbes, using a platinum needle, with the other pole on the gum.

*Dr. Storey*—Asked whether the pus was supposed to enter the blood by direct absorption, or was taken up through the stomach and digestive process.

*Dr. Porre*—Replied that the point was left open for the Section to decide. It would vitiate the blood in either or in both ways, or if the individual had sufficient vitality he might throw off the pus and resist its effects for an indefinite time, perhaps for years, but he must eventually yield. Extraction was resorted to only when the tooth was past salvation.

*Dr. Barrett*—Thought the discussion was not up to the standard of medical knowledge which should be exhibited in such an assemblage as this. He objected to the term "pyogenic membrane" as savoring of long ago exploded theories. In the consideration of pus centres we could not ignore the later advances in pathological knowledge, nor refuse to consider the influence of microbes. Wherever there was pus there was infection, and all treatment must be based upon antisepsis. He could not subscribe to the idea of wandering pus-corpuscles, nor that the swallowing of pus necessarily induced pyæmia.

On motion the subject was passed.

(TO BE CONTINUED.)

