# Correction

**Published:** 1887-11

**Authors:** 


CORRECTION.

In the report of the International Medical Congress in the October number, page 535, Dr. Younger is said to have recommended the use of *Nitric* acid for necrosed bone. It should read *Lactic* acid.

